# Natural Polyphenols for Prevention and Treatment of Cancer

**DOI:** 10.3390/nu8080515

**Published:** 2016-08-22

**Authors:** Yue Zhou, Jie Zheng, Ya Li, Dong-Ping Xu, Sha Li, Yu-Ming Chen, Hua-Bin Li

**Affiliations:** 1 Guangdong Provincial Key Laboratory of Food, Nutrition and Health, School of Public Health, Sun Yat-sen University, Guangzhou 510080, China; zhouyue3@mail2.sysu.edu.cn (Y.Z.); zhengj37@mail2.sysu.edu.cn (J.Z.); liya28@mail2.sysu.edu.cn (Y.L.); xudp@mail2.sysu.edu.cn (D.-P.X.); chenyum@mail.sysu.edu.cn (Y.-M.C.); 2 School of Chinese Medicine, The University of Hong Kong, Hong Kong, China; u3003781@connect.hku.hk; 3 South China Sea Bioresource Exploitation and Utilization Collaborative Innovation Center, Sun Yat-sen University, Guangzhou 510006, China

**Keywords:** polyphenol, flavonoid, anticancer, antioxidant, anti-inflammation

## Abstract

There is much epidemiological evidence that a diet rich in fruits and vegetables could lower the risk of certain cancers. The effect has been attributed, in part, to natural polyphenols. Besides, numerous studies have demonstrated that natural polyphenols could be used for the prevention and treatment of cancer. Potential mechanisms included antioxidant, anti-inflammation as well as the modulation of multiple molecular events involved in carcinogenesis. The current review summarized the anticancer efficacy of major polyphenol classes (flavonoids, phenolic acids, lignans and stilbenes) and discussed the potential mechanisms of action, which were based on epidemiological, in vitro, in vivo and clinical studies within the past five years.

## 1. Introduction

Globally, there were approximately 14.1 million new cancer cases in 2012, and the number was estimated to reach 25 million in 2032. Aside from the high incidence, cancer is also one of the leading causes of death. In 2012 alone, there were about 8.2 million cancer-related deaths, which were mainly attributed to lung, gastric, colorectal, liver, breast, prostate and cervical cancer [1]. The situation urges the research of cancer prevention and treatment. In the last two decades, the anticancer effects of natural polyphenols have become a hot topic in many laboratories. Meanwhile, polyphenols are potential candidates for the discovery of anticancer drugs. Polyphenols are defined as compounds having at least one aromatic ring with one or more hydroxyl functional groups attached. Natural polyphenols refer to a large group of plant secondary metabolites ranging from small molecules to highly polymerized compounds [2]. Polyphenols are widely present in foods and beverages of plant origins (e.g., fruits, vegetables, spices, soy, nuts, tea and wine) [3,4,5]. Based on chemical structures, natural polyphenols can be divided into five classes, including flavonoids, phenolic acids, lignans, stilbenes and other polyphenols. Flavonoids and phenolic acids are the most common classes, and account for about 60% and 30% of all natural polyphenols, respectively (Table 1) [6]. A plethora of studies have documented the anticancer effects of natural polyphenols [7,8,9,10,11]. Noteworthy examples include anthocyanins from blueberries, epigallocatechin gallate (EGCG) from green tea, resveratrol from red wine and isoflavones from soy. The anticancer efficacy of natural polyphenols has largely been attributed to their potent antioxidant and anti-inflammatory activities as well as their abilities to modulate molecular targets and signaling pathways, which were associated with cell survival, proliferation, differentiation, migration, angiogenesis, hormone activities, detoxification enzymes, immune responses, etc. [12,13].

The present review summarized recent discoveries about the anti-carcinogenic properties of natural polyphenols and discussed the mechanisms of action, which were based on evidence from epidemiological studies, laboratory experiments and clinical trials.

## 2. Epidemiological Studies

Evidence from epidemiological studies is inconsistent, especially when considering the results of prospective cohort studies (Table 2). A case-control study in Canada reported favorable effects of a high dietary intake of total flavonoids on lung cancer risks [14]. Apart from this, in a Korean study, for women, the intake of total flavonoids, as well as flavones and anthocyanidins, was inversely associated with the risk of gastric cancer [15]. However, another study in America found no significant association between flavonoids intake and the incidence or survival of gastric cancer [16]. For colorectal cancer, a meta-analysis showed protective roles of high dietary isoflavone intake [17]. Besides, a Spanish case-control study suggested that the dietary intake of total flavonoids (especially certain subclasses) and lignans might decrease colorectal cancer risks [18]. However, large prospective cohorts showed that high habitual consumption of flavonoids could not protect against colorectal cancer [19]. In addition, the Fukuoka study reported no association between total dietary polyphenols and colorectal cancer risks [20]. For hepatocellular carcinoma (HCC), the European Prospective Investigation into Cancer and Nutrition suggested that a high intake of dietary flavanols, but not total flavonoids, might modestly decrease HCC risks [21,22]. In addition, according to a meta-analysis, the risk of breast cancer was reduced in women with a high intake of flavonols and flavones [23]. Studies also suggested that soy isoflavone intake reduced breast cancer risk for Asian women, which was more potent for post-menopausal women (OR 0.46, 95% CI 0.28–0.78) than for premenopausal women (OR 0.63, 95% CI 0.50–0.80). However, for women in Western countries, no significant association could be found, which might due to low levels of isoflavone consumption in the Western population [24,25]. In addition, the estrogen receptor (ER) status might modify the association. For example, a U.S. prospective cohort study showed that a modest inverse trend existed for dietary flavanols intake and the risk of ER-negative breast cancer, but not ER-positive cancer [26]. For prostate cancer, data from a Netherlands cohort study showed that dietary flavonoid intake was correlated with decreased risks of advanced stage prostate cancer but not overall or non-advanced prostate cancer [27]. On the contrary, in a prospective cohort study, the intake of total flavonoids as well as flavan-3-ols, isoflavones, and proanthocyanidins, increased prostate cancer risks [28].

It should be noted that the assessment of polyphenol intakes in many epidemiological studies was based on food questionnaires, which could not provide the exact composition of foods. Therefore, it might be difficult for them to reflect the real impact of natural polyphenols on cancer. In this case, the experimental study in cell culture or animal modes might be a more direct way to assess the anticancer efficacy of natural polyphenols as well as to examine the possible mechanisms involved in this process.

## 3. Experimental Studies

Accumulating evidence from laboratory studies has supported the anticancer properties of natural polyphenols. Given the vast number of studies, a search of PubMed and Web of Science was conducted to identify relevant peer-reviewed articles published in English within 5 years.

### 3.1. Anthocyanins

**Figure 1:**
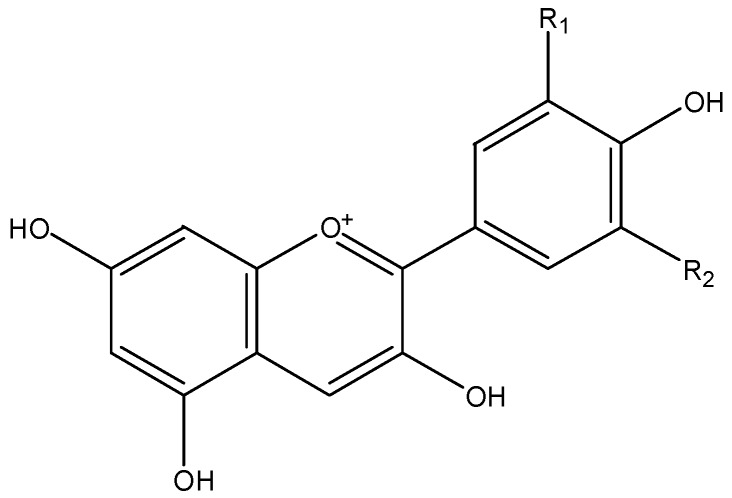
The chemical structures of cyanidin (R_1_ = OH, R_2_ = H), delphinidin (R_1_ = R_2_ = OH), peonidin (R_1_ = OCH3, R_2_ = H), petunidin (R_1_ = OCH3, R_2_ = OH), pelargonidin (R_1_ = R_2_ = H) and malvidin (R_1_ = R_2_ = OCH3).

Anthocyanins (Figure 1), which occur ubiquitously throughout the plant kingdom, are the basis for the bright attractive red, blue and purple colors of fruits and vegetables. In plants, anthocyanins are usually glycosylated with glucose, galactose, arabinose, rutinose, etc. The aglycone forms are known as anthocyanidin, including cyanidin, delphinidin, peonidin, petunidin, pelargonidin, and malvidin [29].

Among anthocyanins, delphinidin possesses strong anticancer activities. Studies have shown that delphinidin treatment induced apoptosis and cell cycle arrest in several types of cancer. This effect might be due to suppression of the NF-κB pathway [30,31]. The over-expression of human epidermal growth factor receptor 2 (HER2) is usually associated with poor prognosis. A study found that two anthocyanins extracted from black rice, peonidin-3-glucoside and cyaniding-3-glucoside, could induce apoptosis and selectively decrease cell proliferation and tumor growth of HER2 positive breast cancer [32]. In addition, peonidin-3-glucoside treatment significantly suppressed invasion and metastasis of lung cancer cells by down-regulating the matrix metalloproteinase (MMP) [33]. In similar ways, cyanidin-3-*O*-sambubioside from *Acanthopanax sessiliflorus* fruit inhibited angiogenesis and invasion of breast cancer cells [34]. Though anthocyanins are usually considered as antioxidants, a study showed that certain anthocyanins (cyanidin and delphinidin) exhibited oxidative stress-based cytotoxicity to colorectal cancer cells [35]. Another study evaluated the impact of chemical structures on chemopreventive activities of anthocyanins in colon cancer cells. Data indicated that nonacylated monoglycosylated anthocyanins were more potent in inhibiting cancer cell growth, while anthocyanins with pelargonidin aglycone and triglycosylation were weak [36]. On the other hand, it was suggested that a mixture of different anthocyanins might be better than a single one in cancer treatment. For example, a combination of sub-optimal concentration of anthocyanidins synergistically suppressed the growth of lung cancer cells. Meanwhile, in a mice model of lung cancer, a mixture of anthocyanidins from bilberry (0.5 mg/mouse) or delphinidin (1.5 mg/mouse) all inhibited tumor growth, and the effective concentration of delphinidin in the mixture was eight-fold lower than the purified compound [7].

### 3.2. Xanthohumol

**Figure 2:**
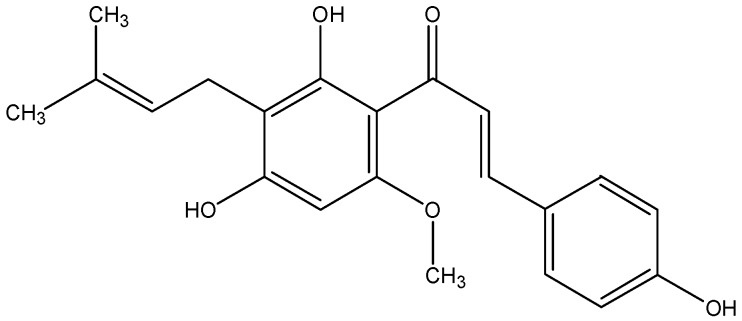
The chemical structure of xanthohumol.

Xanthohumol (Figure 2) is a major prenylated chalcone isolated from hops (*Humulus lupulus*). The compound can also be found in beer, but to a much less extent. In some cancers, the xanthohumol-induced cell death was accompanied by apoptosis and S phase cell cycle arrest [37,38]. A study suggested that the apoptosis induced by treatment of xanthohumol (10–40 μM) to HepG2 liver cancer cells was due to modulation of the NF-κB/p53 signaling pathway [39]. Another study reported that xanthohumol treatment (>5 μM) mediated anticancer activity in human liver cancer cells through suppression of the Notch1 signaling pathway [40]. In addition, xanthohumol could block the estrogen signaling pathway. By doing so, it selectively suppressed the growth of ERα-positive breast cancer both in vitro and in vivo [41]. Cysteine X Cysteine chemokine receptor 4 (CXCR4) is over-expressed in many cancers and mediates metastasis of cancer cells to sites expressing its cognate ligand CXCL12. A study demonstrated that xanthohumol treatment dose- and time-dependently decreased expression of CXCR4, thus inhibiting cell invasion induced by CXCL12 in breast and colon cancer cells [42]. In another study, by promoting production of reactive oxygen species (ROS), xanthohumol treatment inhibited the progression of advanced tumor and the growth of poorly differentiated prostate cancer in the transgenic mice [43].

### 3.3. Flavanols

Flavanols, also known as flavan-3-ols, have the most complex structures among subclasses of flavonoid. Flavanols include simple monomers (catechins) as well as oligomers and polymers, the latter two are known as proanthocyanidins or condensed tannins. Flavanols can be commonly found in foodstuffs [29].

#### 3.3.1. EGCG

**Figure 3:**
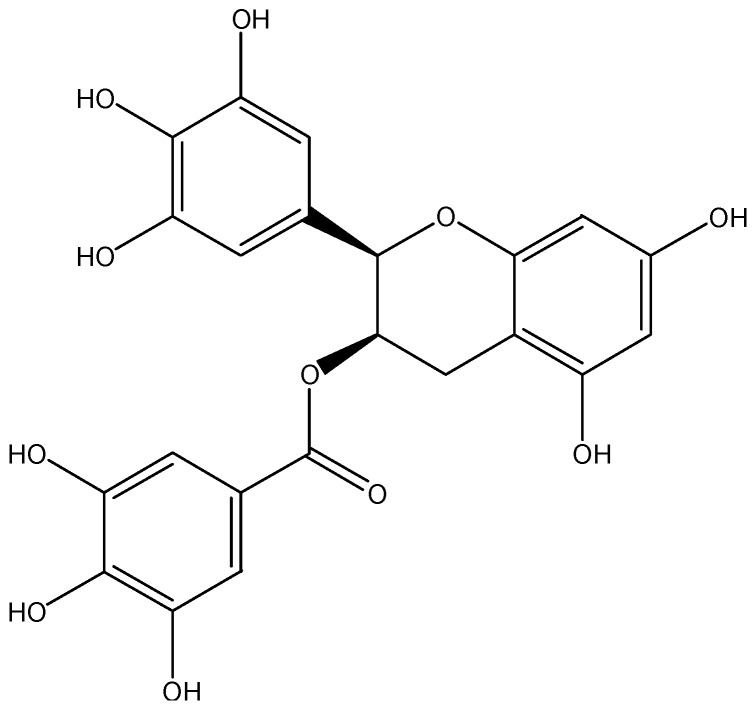
The chemical structure of EGCG.

Smoking is a well-established risk factor of lung cancer. A study showed that EGCG (Figure 3) treatment suppressed nicotine-induced migration and invasion of A549 lung cancer cells in vitro as well as in mice through inhibiting angiogenesis and epithelial-mesenchymal transition (EMT) [9]. The effects of EGCG varied with dose. In CL1-5 lung cancer cells, at concentration of 5–20 μM, EGCG effectively suppressed the invasion and migration through suppressing MMP-2 expression. While at higher concentration (>20 μM), it exhibited anti-proliferation activities through induction of G_2_/M cell cycle arrest but not apoptosis [44]. Another study found that several gastric cancer cell lines were sensitive to EGCG (100 μM) induced apoptosis due to inhibition of survivin, a potent anti-apoptotic protein [45]. Many signaling pathways might be affected by EGCG treatment. A study showed that EGCG (20 μM) exerted anti-proliferative effects in gastric cancer cell by preventing the β-catenin oncogenic signaling pathway [46]. Another study on colon cancer suggested that the Akt, extracellular signal-related kinase (ERK) 1/2 and alternative p38MAPK signaling pathways were involved in the chemopreventive effects of EGCG [47]. Besides, there is a growing interest in cancer epigenetics in recent years mainly due to the reversibility of epigenetic alterations. Major epigenetic alterations involve DNA methylation, histone modifications and miRNAs [48]. The combination of EGCG and sodium butyrate inhibited DNA methytransferases and class I histone deacetylases (HDACs) in colorectal cancer cells, thus modulating global DNA methylation and histone modifications [49]. In addition, the cancer stem cell plays a key role in chemoresistance and recurrence. Both in vitro and in vivo studies showed that EGCG could suppress cancer stem cell growth of colorectal cancer as well as breast cancer [50,51]. The anticancer activities of EGCG might involve modulation of hormone activities. It is known that exposure to estrogen is an important risk factor of breast cancer. A study found that EGCG (1 μM) could suppress estrogen (estradiol, E2)-induced breast cancer cell proliferation [52]. In addition, EGCG treatment down-regulated ERα in ER^+^/PR^+^ breast cancer cells [53]. Treatment of EGCG (20 μM) also inhibited metastasis of breast cancer cells by restoring the balance between MMP and the tissue inhibitor of matrix metalloproteinase (TIMP). Mechanistic studies suggested that the epigenetic induction of TIMP-3 was a key event in this process, which involved modifying the enhancer of zeste homolog 2 and HDAC1 [54]. Androgen deprivation is a main therapy for prostate cancer. It was reported that EGCG could functionally antagonize androgen, leading to suppression of prostate cancer growth both in vitro and in vivo [55].

#### 3.3.2. Procyanidins

A study suggested that procyanidin C1 from Cinnamomi cortex might be able to prevent TGF-β-induced EMT in the A549 lung cancer cells [56]. Another study found that hexmer form of procyanidins from cocoa inhibited the proliferation (50 and 100 μM), induced apoptosis and G_2_/M cell cycle arrest in several colorectal cancer cells, which was possibly mediated by the Akt pathway [57]. Procyanidins from Japanese quince also showed pro-apoptotic effects on Caco-2 colon cancer cells, with the oligomer enriched extract showing a more potent pro-apoptotic activity [58]. Besides, data shows that in breast cancer cells, treatment of procyanidins from evening primrose (25–100 μM gallic acid equivalents) decreased cell viability by promoting apoptosis and reduced cell invasion by suppressing angiogenesis propensity [59].

### 3.4. Flavanones

**Figure 4:**
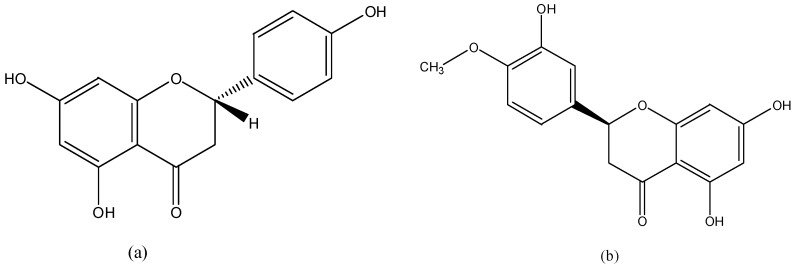
The chemical structures of naringenin (**a**) and hesperetin (**b**).

Flavanones (Figure 4) are abundant in citrus fruits, especially the solid parts of fruit. Major flavanones are naringenin from grapefruit and hesperetin from oranges [2].

#### 3.4.1. Naringenin

In A549 lung cancer cells, naringenin treatment enhanced TRAIL-mediated apoptosis by up-regulating the expression of death receptor 5 [60]. Besides, in SGC-7901 gastric cancer cells, naringenin treatment inhibited cancer cell proliferation, invasion, and migration and induced apoptosis, which might be related to its inhibition of the Akt signaling pathway [61]. Another study in colon cancer cells suggested that the pro-apoptotic activity of naringenin was mediated by the p38-dependent pathway [62]. In HCC cells, naringenin could suppress TPA-induced cancer cell invasion by down-regulating multiple signaling pathways, such as the NF-κB pathway, the ERK and c-Jun N-terminal kinase (JNK) signaling pathway [63]. Besides, naringenin treatment to HepG2 liver cancer cells induced mitochondrial-mediated apoptosis and cell cycle arrest through up-regulation of p53 [64]. In breast cancer cells, naringenin demonstrated anti-estrogenic activity in estrogen-rich status and estrogenic activity in estrogen-deficient status [65]. In addition, oral administration of naringenin suppressed breast cancer metastases after surgery by modulating the host immunity [66]. 

#### 3.4.2. Hesperetin

In gastric cancer cells, hesperetin treatment (100–400 μM) decreased cell proliferation and induced mitochondria-mediated apoptosis via promoting intracellular ROS accumulation. Meanwhile, the compound (i.p. 20–40 mg/kg thrice a week) significantly suppressed the growth of xenograft tumors in mice model of gastric cancer [67]. Besides, dietary hesperetin showed anti-proliferative activities against chemical-induced colon carcinogenesis. Oral supplements of hesperetin (20 mg/kg/day) reduced the proliferating cell nuclear antigen, the formation of aberrant crypt foci induced by 1,2-dimethylhydrazine in rat [68]. In breast cancer cells, hesperetin (40–200 μM) induced growth inhibition also involved mitochondria-mediated apoptosis, increased ROS and activation of ASK1/JNK pathway [69]. Cancer cells usually have high levels of glucose uptake and metabolism, which plays an important role in tumor growth. A study suggested that the anti-proliferative effects of hesperetin (50–100 μM) on breast cancer were possibly due to the suppression of glucose uptake [70]. Another study found that hesperetin treatment (IC_50_ 40–90 μM) decreased proliferation and induced apoptosis in PC-3 prostate cancer cells, which was likely mediated by inhibition of the NF-κB pathway [71]. In addition, hesperetin (IC_50_ 650 μM) exhibited potential anticancer effects on cervical cancer cells through the induction of both extrinsic and intrinsic apoptosis [72]. 

### 3.5. Flavones

**Figure 5:**
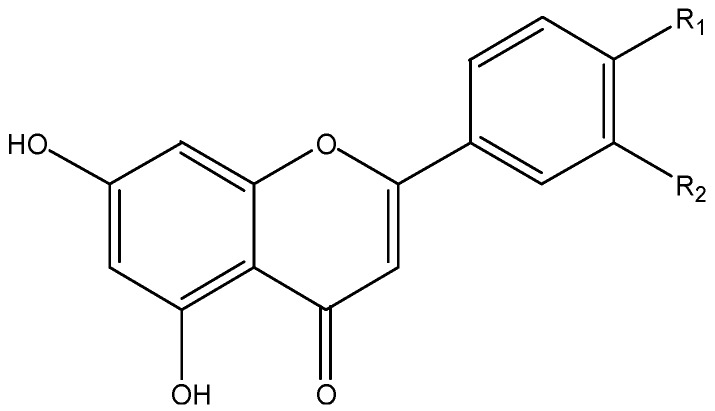
The chemical structures of apigenin (R_1_ = OH, R_2_ = H), chrysin (R_1_ = R_2_ = H) and luteolin (R_1_ = R_2_ = OH).

Flavones (Figure 5) in food are usually the glycosides of apigenin and luteolin. Important dietary sources of flavones are parsley and celery [2].

#### 3.5.1. Apigenin

Apigenin is a common flavonoid widely distributed in plant-based food, such as orange, parsley, onions, tea and wheat sprouts [73]. In H460 lung cancer cells, treatment of apigenin (40–160 μM) induced apoptosis and DNA damage, which was accompanied by increased production of ROS and Ca^2+^ as well as a change of the Bax/Bcl-2 ratio [74]. Apigenin (20 μg/mL) also induced apoptosis in gastric cancer cells, especially in the undifferentiated gastric cancer cells, while showed little cytotoxicity to normal gastric cells [75]. *Helicobacter pylori* infection is known to cause ulcers and is possibly linked to gastric cancer. Atrophic gastritis was suggested to be a critical step in *Helicobacter pylori*-induced carcinogenesis. A study found that apigenin administration (30–60 mg/kg/week) could prevent *Helicobacter pylori*-induced atrophic gastritis as well as gastric cancer development in Mongolian gerbils [76]. Additionally, apigenin treatment (20–120 μM) suppressed proliferation, invasion and migration of several colorectal cancer cell lines. The compound (50 mg/kg) also inhibited tumor growth and metastasis in the orthotopic colorectal cancer model [77]. 

About 20% of breast cancer cases are HER2-positive, with amplification of human epidermal growth factor receptor (HER2) or over-expression of HER2 protein. These cancers are usually more aggressive and more resistant to hormone treatment than other types of breast cancer. A study found that apigenin treatment (20–100 μM) significantly suppressed growth and caused apoptosis in HER2-positive breast cancer cells, which was possibly mediated by inhibition of the signal transducer and activator of transcription 3 (STAT3) signaling pathway [78]. Another study reported anticancer effects of apigenin on MDA-MB-231 breast cancer cells in vitro (10–40 μM) and in vivo (5 and 25 mg/kg). Possible mechanisms included induction of G_2_/M cell cycle arrest and epigenetic alterations. Apigenin inhibited HDACs, which induced acetylation of histone H3 in the p21^WAF1/CIP1^ promoter region, leading to enhanced transcription of p21^WAF1/CIP1^ [79]. Similar epigenetic effects were also found in prostate cancer. Apigenin inhibited HDACs, especially HDAC1 and HDAC3 expression. In this way apigenin treatment (20–40 μM) induced cell cycle arrest and apoptosis in prostate cancer cells and markedly inhibited tumor growth in mice (oral administration: 20 and 50 μg/mouse/day) [80]. In addition, apigenin treatment to mice (20 and 50 μg/mouse/day) markedly decreased tumor volumes of the prostate, inhibited angiogenesis and completely prevented distant organ metastasis, which at least in part, was mediated by the PI3K/Akt/Forkhead box O (FoxO) signaling pathway [81].

#### 3.5.2. Chrysin

Chrysin is a naturally occurring flavone present in honey and propolis as well as the passion flower (*Passiflora caerulea*), and has displayed a variety of bioactivities, such as antioxidant, anti-inflammatory and anticancer activities [82]. AMPK activation is associated with cancer cell apoptosis. A study suggested that AMPK activation might be involved in the growth inhibition and apoptosis induced by chrysin treatment (10 μM) in lung cancer cells, and ROS might be a key regulator in this process [83]. Chrysin (50–100 μM) also exhibited chemopreventive effects in colorectal cancer cells, mainly as a result of TNF-mediated apoptotic cell death, and the aryl hydrocarbon receptor, a transcriptional factor, seemed to modulate this process [84]. Besides, in human triple-negative breast cancer cells, chrysin treatment (5, 10 and 20 μM) dose-dependently inhibited the potential of cancer cells to invasion and migration by down-regulating MMP-10, EMT and the PI3K/Akt signaling pathway [82]. 

#### 3.5.3. Luteolin

Luteolin is abundant in artichoke as well as several spices, including sage, thyme and oregano. In A549 lung cancer cells, luteolin exhibited significant cytotoxic effects (IC_50_ 40.2 μM) through induction of G_2_ cell cycle arrest and apoptosis. The apoptosis was induced in a mitochondria-dependent pathway and was associated with activation of JNK and inhibition of NF-κB (p65) translocation [85]. The micro-environment around cancer cells is highly involved in cancer progression. It was reported that luteolin (1–10 μM) effectively suppressed IL-4 induced polarization of tumor-associated macrophages (major components of cancer cell micro-environment) and consequently inhibited monocyte recruitment and migration of Lewis lung cancer cells [86]. Hypoxia is another important component of cancer micro-environment. In non-small lung cancer cells, high levels of hypoxia are usually related to EMT. Luteolin treatment (5–50 μM) to non-small lung cancer cells could inhibit hypoxia-induced EMT as well as cell viability, proliferation and motility. The effect was at least partly through suppressing the expression of integrin β1 and FAK [87]. More importantly, luteolin administration (i.p. 10 and 30 mg/kg/day) effectively suppressed tumor growth in a lung cancer mice model with EGF receptor mutation and drug resistance [88].

In a human gastric cancer xenograft model, luteolin treatment (i.p. 10 mg/kg/day) significantly suppressed tumor growth, without causing apparent toxicity or weight loss [89]. Luteolin treatment (20–100 μM) also exhibited cytotoxic effect on several colon cancer cell lines through induction of apoptosis and cell cycle arrest. Meantime, the same treatment exerted no evident toxicity on normal differentiated enterocytes [90,91]. These effects of luteolin might be associated with down-regulation of the IGF-1-mediated PI3K/Akt and ERK1/2 pathways, and suppression of synthesis of sphingosine-1-phosphate and ceramide traffic [90,91]. Besides, it was indicated that ERα was a possible target of luteolin. By down-regulating the expression of ERα, luteolin treatment (10–40 μM) suppressed IGF-1-mediated PI3K/Akt pathway, leading to growth inhibition of MCF-7 breast cancer cells accompanied by cell cycle arrest and apoptosis [92]. In the MDA-MB-231 ER-negative breast cancer cells, luteolin treatment also induced cell cycle arrest and apoptosis possibly mediated by EGFR. In addition, luteolin-supplemented diet (0.01% or 0.05%) effectively reduced tumor burden in mice inoculated with MDA-MB-231 cells [93]. Besides, in LNCaP prostate cancer cells, luteolin treatment (30 μM) arrested the cell cycle at G_1_/S phase, induced cell apoptosis and inhibited cell invasion. The possible mechanism might be down-regulated expression of prostate-specific antigen by luteolin [94]. 

### 3.6. Flavonols

**Figure 6:**
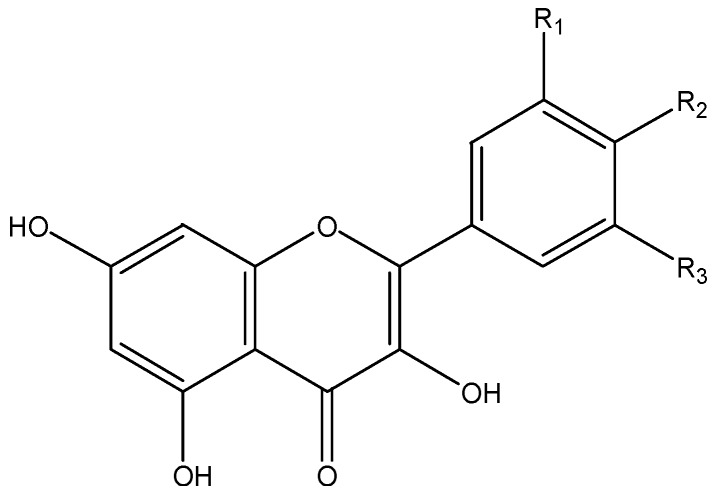
The chemical structures of quercetin (R_1_ = H, R_2_ = R_3_ = OH), kaempferol (R_1_ = R_3_ = H, R_2_ = OH), myricetin (R_1_ = R_2_ = R_3_ = OH), galangin (R_1_ = R_2_ = R_3_ = H) and isorhamnetin (R_1_ = H, R_2_ = OH, R_3_ = OCH3).

Flavonols (Figure 6) are probably the most widely distributed flavonoids in foods, but they are usually present at relatively low concentrations [2]. Representatives of this subclass are quercetin, kaempferol, myricetin, galangin and isorhamnetin.

#### 3.6.1. Quercetin

Quercetin treatment (IC_50_ 2.30 ± 0.26 μM) to A549 lung cancer cells induced growth inhibition via apoptosis. In similar ways, quercetin (8.4 mg/kg) inhibited the growth of transplanted lung cancer in nude mice [95]. On the other hand, though exposure of gastric cancer cells to quercetin (IC_50_ 40 and 160 μM in two cell lines respectively) led to pronounced apoptosis, the treatment also induced protective autophagy, which impaired the anticancer effects of quercetin [96]. AMPK-mediated signaling pathway, which participates in regulation of energy homeostasis, is important for the adaptive responses of cancer cells and might be critical for the effects of quercetin. A study found that quercetin treatment (i.p. 50 mg/kg/day) significantly decreased tumor volume in the HCT116 colon cancer xenograft model by reducing AMPK activity. Similarly, by inhibiting AMPK, the apoptosis induced by quercetin (100 μM) was more pronounced under hypoxic conditions than normoxic conditions in HCT116 colon cancer cells [97]. Besides, in a mouse model of colorectal cancer, dietary quercetin supplementation (25 mg/kg/day) alleviated several symptoms of cachexia such as body weight, grip strength and muscle mass [98]. Another study found that quercetin treatment (0.05–0.15 mM) to HCC cells effectively inhibited proliferation and induced apoptosis through up-regulation of Bad and Bax, and concomitant down-regulating Bcl-2 and survivin. Importantly, quercetin (i.p. 40 mg/kg/day) also exhibited excellent inhibition effects on tumor growth in mice [99]. 

The exposure of MCF-7 breast cancer cells to quercetin (50–200 μM) caused a dose- and time-dependent decrease of proliferation through induction of apoptosis, which was accompanied by up-regulation of Bax and down-regulation of Bcl-2 [100]. The inhibition of insulin receptor signaling by quercetin (100 μM) also impairs proliferation of MDA-MB-231 breast cancer cells. Quercetin feeding (50 μg/mouse/day) resulted in a significant decrease of tumor growth in mice model of breast cancer [101]. In another study, quercetin (1–100 μM) inhibited breast cancer cells growth and migration via reversing EMT, which was linked with the modulation of β-catenin as well as its target genes (e.g., cyclin D1 and c-Myc) [102]. VEGFR2-mediated pathway participates in the angiogenesis in cancer development. Quercetin (34 mg/kg/day) inhibited angiogenesis of breast cancer xenograft in mice, which was performed through suppressing this pathway [103]. Besides, dietary quercetin (200 mg/kg body weight thrice a week) protected against prostate carcinogenesis induced by hormone (testosterone) and carcinogen (*N*-methyl-*N*-nitrosourea) in rats [104]. In another preclinical rat model of prostate cancer, oral administration of quercetin (200 mg/kg/day) prevented cancer development by down-regulating the cell survival, proliferative and anti-apoptotic proteins [105]. In HeLa cervical cancer cells, quercetin treatment (110.38 ± 0.66 μM) led to ROS accumulation to induce apoptosis and G_2_/M cell cycle arrest [106]. 

#### 3.6.2. Kaempferol

Kaempferol is a natural flavonol broadly distributed in apples, strawberries, broccoli and beans, and exhibits a wide range of beneficial properties, such as cardioprotective, anti-diabetic, and anti-allergic effects [107]. In A549 lung cancer cells, kaempferol treatment inhibited TGF-β1-induced EMT and migration through suppressing the phosphorylation of smad3 mediated by Akt1 [107]. Another study reported that kaempferol treatment exhibited significant anti-proliferative effects on MKN28 and SGC7901 gastric cancer cells without apparent cytotoxicity to normal gastric epithelial cells. The possible mechanism might be induction of apoptosis and G_2_/M cell cycle arrest. More importantly, administration of kaempferol suppressed gastric cancer growth in vivo [108]. In HT-29 colon cancer cells, the treatment of kaempferol (0–60 μM) provoked apoptosis by activating the death receptor pathway and mitochondrial pathway [109]. Another study in SK-HEP-1 human liver cancer cells found G_2_/M cell cycle arrest and autophagy following kaempferol treatment, which might be the result of the modulation of CDK1/cyclin B expression and AMPK and AKT signaling pathways [110]. Kaempferol induced apoptosis in MCF-7 breast cancer cells [111]. In the same cell line, treatment of kaempferol (100 μM) also significantly suppressed glucose uptake mediated by GLUT1, which might be another mechanism underlying its anti-proliferative effects [112]. Besides, both in vitro and in vivo study revealed that kaempferol could prevent breast cancer induced by 17β-estradiol or triclosn, an exogenous estrogen [113]. Kaempferol treatment also inhibited breast cell invasion through down-regulating the expression and activity of MMP-9 by blocking the PKCδ/MAPK/AP-1 cascades [114].

#### 3.6.3. Myricetin

Myricetin is rich in berries, walnuts and herbs. Myricetin treatment to gastric cancer cells exhibited anti-proliferative effects by inducing apoptosis and cell cycle arrest [115]. In HCT-15 human colon cancer cells, myricetin treatment induced apoptotic cell death by modulating the Bax/Bcl-2-dependent pathway [116]. Similarly, myricetin also decreased the expression of anti-apoptotic survivin and Bcl-2 and increased the expression of pro-apoptotic Bax in HCC cells and in vivo [117]. 

#### 3.6.4. Galangin

Galangin is a naturally occurring flavonoid rich in oregano as well as in *Alpinis officinarum*, a common spice in Asia. Galangin treatment (50–200 μM) to SNU-484 human gastric cancer cells dose- and time-dependently inhibited cell proliferation through induction of apoptosis [118]. Besides, in hepG2 liver cancer cells, galangin treatment (10–30 μM) significantly inhibited chemical-induced cell invasion and metastasis by modulating the PKC/ERK pathway [119]. Another study suggested that galangin (79.8–134 μM) could promote ER stress to suppress the proliferation of HCC cells [120].

#### 3.6.5. Isorhamnetin

Isorhamnetin is a natural flavonoid rich in fruits and vegetables as well as tea, and is also an immediate metabolite of quercetin, which has drawn attention for its excellent anti-inflammatory and anticancer activities [121,122]. 

Treatment of isorhamnetin to A549 lung cancer cells induced apoptotic cell death, which was accompanied by the up-regulation of capase-3, Bax, p53 and the down-regulation of Bcl-2, cyclin D1 and PCNA protein. More importantly, isorhamnetin administration to tumor-bearing mice significantly suppressed tumor growth [123]. Additionally, isorhamnetin suppressed gastric cancer proliferation and invasion, and induced apoptosis by modulating the peroxisome proliferator-activated receptor γ (PPAR γ)-mediated pathway in vitro and in vivo [124]. Another study investigated the anti-proliferative activity of isorhamnetin in several human colorectal cancer cell lines (HT29, HCT116 and SW480), and found that the compound inhibited proliferation of all tested cancer cells by blocking the PI3K/Akt/mTOR pathway [125]. Both in vitro and in vivo experiments suggested that the anticancer property of isorhamnetin in colon cancer involved inhibition of inflammation as well as oncogenic Src activity and consequential loss of nuclear β-catenin [126]. Another study documented the anti-proliferative and pro-apoptotic activities of isorhamnetin in breast cancer cells, which was probably mediated by the Akt and MAPK kinase signaling pathways [121]. Besides, in MDA-MB-231 breast cancer cells, isorhamnetin treatment significantly suppressed cell invasion by down-regulating MMP-2 and MMP-9, which might be associated with the inhibition of p38 MAPK and STAT3 [122].

### 3.7. Isoflavones

**Figure 7:**
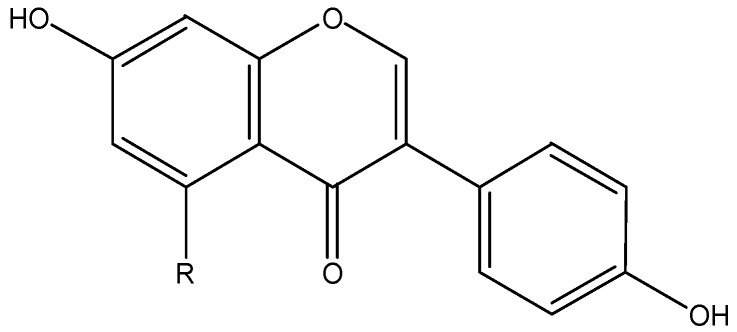
The chemical structures of daidzein (R = H) and genistein (R = OH).

Due to structural similarities to estrogen, isoflavones (Figure 7) have been classified as phytoestrogen, another important class of phytochemicals. Genistein and daidzein from soy are representative members of this subclass [2].

#### 3.7.1. Daidzein

Data indicated that daidzein was an apoptosis inducer in liver cancer cells and treatment of daidzein (200–600 μM) caused mitochondrial-dependent apoptosis mediated by the Bcl-2 family [127]. In an in vitro study, daidzein (50 μM) as well as its metabolites R-equol and S-equol, suppressed the invasion of MDA-MB-231 human breast cancer cells at least partly through the down-regulation of MMP-2 expression [128]. However, another study reported that daidzein treatment (3–10 μM) up-regulated proto-oncogene BRF2 in ER-positive breast cancer cells but not ER-negative cells. Female mice treated with a high-isoflavone commercial diet showed significantly increased BRF2 expression [129]. 

#### 3.7.2. Genistein

Genistein is the most abundant isoflavonoid contained in soy as well as soy products and is also a major active component of hormonal supplements for menopausal women [10]. In H446 lung cancer cells, genistein treatment (25–75 μM) effectively suppressed the cell proliferation and migration, which was accompanied by induction of apoptosis and G_2_/M cell cycle arrest. Importantly, the treatment also suppressed the expression of Forehead box protein M1 and its target genes regulating cell cycle or apoptosis, such as survivin, cyclin B1 and Cdc25. Therefore, the effects of genistein were at least partly mediated by Forkhead box protein M1 [130]. In addition, genistein treatment (15 μM) to gastric cancer cells suppressed the cancer cell stem-like abilities, includingself-renewal, drug resistance and carcinogenicity, which might be due to down-regulation of stemness related genes as well as drug resistance gene ABCG2. Meantime, genistein (i.p. 1.5 mg/kg/day) significantly decreased the weight and size of gastric cancer inoculated in nude mice [131]. Besides, genistein (25–100 μM) exhibited anti-proliferative and pro-apoptotic effects on colon cancer cells. The study indicated that inhibition of oncogenic miR-95, Akt and SGK as well as phosphorylation of Akt could be involved in these anticancer effects. Moreover, genistein treatment (i.p. 20, 50, 80 mg/kg/day) to mice significantly decreased the weight and size of transplanted colorectal cancer [132]. Oral administration of genistein also inhibited angiogenesis and suppressed metastasis of colorectal cancer to distant organs in mice [133]. According to in vitro studies, the anticancer effects of genistein on colorectal cancer might involve the suppression of Wnt, NF-κB signaling pathways [134,135]. Additionally, in nude mice inoculated with liver cancer cells, oral administration of genistein (50 mg/kg/day) significantly suppressed the intrahepatic metastasis [136].

Genistein treatment (5, 10 or 20 μM) elicited growth inhibition of MDA-MB-231 breast cancer cells, which was accompanied by apoptosis and G_2_/M cell cycle arrest. This effect might be mediated by down-regulation of the NF-κB activity via the Notch-1 pathway [137]. In MCf-7 breast cancer cells, genistein treatment (15 and 30 μM) also inhibited cell growth, induced apoptosis and decreased the CD44^+^CD24^−^ cancer stem cells. Importantly, genistein (i.p. 20 and 50 mg/kg/day) could also target breast cancer stem cells to reduce the volume and weight of xenograft tumors in nude mice. The effects might be correlated with down-regulation of Hedgehog-Gli1 signaling pathway [138]. However, some studies found that genistein has adverse effects on breast cancer treatment. A study suggested that the ERα/ERβ ratio could be a determinant of genistein functions in breast cancer. In breast cancer with a low ERα/ERβ ratio (e.g., T4D7 cells), genistein treatment might be harmless or even beneficial, while in breast cancer with a high ratio (e.g., MCF-7 cells), the treatment might be counterproductive [139]. Genistein (10 μM) could also affect the expression and function of ATP-binding cassette drug transporters in breast cancer cells. The effect resulted in an increase of efflux and resistance of chemotherapeutic drugs (doxorubicin and mitoxantrone) in MCF-7 cells [10]. Moreover, in athymic mice model of breast cancer, a low dose long-term treatment of genistein (≤500 ppm) led to tumor growth as well as more aggressive and advanced phenotypes [140]. Genistein was also reported to have different effects on prostate cancer cells. In LAPC-4 cells with wild androgen receptor, genistein treatment (0.5–50 μM) dose dependently suppressed cell proliferation and androgen receptor. However, in LNCaP cells with T877A mutant androgen receptor, genistein promoted cancer cell growth and androgen receptor at physiological concentration (0.5–5 μM), but showed inhibitory activities at higher concentration. Similar biphasic activities of genistein were also observed in PC-3 cells transfected with androgen receptor mutants [141]. In addition, the exposure of HeLa cervical cancer cells to genistein (IC_50_ 100 μM) led to growth inhibition mediated by apoptosis and G_2_/M cell cycle arrest and suppressed cell migration by modulating MMP-9 and TIMP-1 [142].

### 3.8. Phenolic Acids

**Figure 8:**
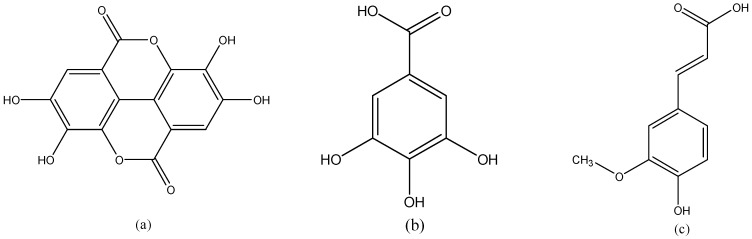
The chemical structures of (**a**) ellagic acid; (**b**) gallic acid and (**c**) ferulic acid.

Phenolic acids (Figure 8) can be mainly classified into two groups, hydroxybenzoic acid and hydroxycinnamic acid. Hydroxybenzoic acids present in few edible plants and are not considered to be of high nutritional interest. The other group is more common in food, but its consumption is highly variable, depending on intake of coffee [2].

#### 3.8.1. Ellagic Acid

Ellagic acid is a dietary flavonoid abundantly in pomegranate, grapes, strawberries and walnuts [143]. Ellagic acid (50–200 μM) exerted anti-proliferative and pro-apoptotic effects in colon cancer cell lines in a concentration dependent manner [144]. Besides, in a chemical-induced liver cancer rat model, oral administration of ellagic acid (30 mg/kg/day) normalized the permeability of mitochondrial outer membrane and alleviated inflammation-mediated cancer cell proliferation [145]. Ellagic acid (10–40 μg/mL) also showed growth inhibitory effects on MCF-7 breast cancer cells, which was accompanied by G_0_/G_1_ cell cycle arrest. The modulation of the TGF-β/Smads signaling pathway was suggested to be the potential mechanism [143]. Furthermore, exposure to ellagic acid (i.p. 50 and 100 mg/kg/day) suppressed tumor growth and angiogenesis in mice implanted with breast cancer cells [146]. In another study, non-cytotoxic dose of ellagic acid (25 and 50 μM) to androgen independent prostate cancer cells markedly suppressed the cell invasion and motility. The effect might be the result of down-regulation of MMPs [147]. Besides, at higher dose (10–100 μM), ellagic acid treatment was found to induce growth inhibition and caspase-dependent apoptosis in PC3 prostate cancer cells in a dose responsive manner [148]. 

#### 3.8.2. Gallic Acid

Gallic acid is widely distributed in plant-based food in free forms as well as part of hydrolyzable tannins. Blackberry, raspberry, walnuts, chocolate, wine, green tea and vinegar are rich sources of the compound. Gallic acid possesses various pharmacological activities, such as anti-microbial, anti-inflammatory and anticancer activities [149,150]. Exposure to gallic acid (3.5 μM) inhibited migration of AGS gastric cancer cells, which was possibly mediated by up-regulation of RhoB as well as down-regulation of AKT/small GTPase signals and NF-κB activity. In addition to this, compared with the control, feeding with gallic acid solution (0.25% and 0.5%) significantly decreased tumor size and weight in mice models of gastric cancer [151]. The ROS-dependent pro-apoptotic effects of gallic acid led to decreased viability of different cancer cells, such as HCT-15 colon cancer cells (200 μM) and LNCaP prostate cancer cells (80 μg/mL) [149,152]. Besides, gallic acid treatment selectively inhibited growth of liver cancer cells through the mitochondria-mediated apoptotic pathways (IC_50_ for cancer cells 28.5 ± 1.6 μg/mL and 22.1 ± 1.4 μg/mL, for normal human hepatocytes 80.9 ± 4.6 μg/mL) [153]. Studies on MCF-7 breast cancer cells also showed that gallic acid treatment inhibited cell proliferation (IC_50_ 80.5 µM) and induced apoptosis via both the extrinsic and intrinsic pathways [150]. Additionally, exposure to gallic acid (25 and 50 μM) suppressed the invasion and migration of PC-3 prostate cancer cells through down-regulation of MMP-2 and MMP-9 [154]. In another study, gallic acid (50, 100, and 200 μM) in PC-3 prostate cancer cells provoked DNA damage and inhibited expression of DNA repair genes, which contributed to gallic-induced growth inhibition [155]. Treatment with gallic acid (10–40 μg/mL) decreased cell viability, proliferation, invasion and angiogenesis HeLa and HTB-35 cervical cancer cells, but showed less cytotoxicity on normal cells (HUVEC), indicating a potential role of the compound in cervical cancer treatment [156]. 

#### 3.8.3. Ferulic Acid

The main dietary sources of ferulic acid are cereal grains, particularly the outer parts of grain. The compound has attracted great attention due to its therapeutic activities against various diseases, such as cancer, cardiovascular and neurodegenerative diseases [157,158].

It was reported that ferulic acid was a pro-oxidant at high concentration or in the presence of metal ions such as copper. Since the increased level of copper was observed in many cancers, and cancer cells are usually under greater oxidative stress than normal cells, the pro-oxidant ability of ferulic acid might lead to selective cytotoxicity to cancer cells [157]. Ferulic acid (10 μg/mL) also decreased cell viability and enhanced efficacy of radiotherapy in two cervical cancer cell lines (HeLa and ME-180), possibly through promotion of ROS [159]. Another study on prostate cancer found that the effects of ferulic acid varied with cell types. Ferulic acid treatment caused cell cycle arrest in PC-3 cells (IC_50_ 300 μM), and led to apoptosis in LNCaP cells (IC_50_ 500 μM) [158].

### 3.9. Lignans

**Figure 9:**
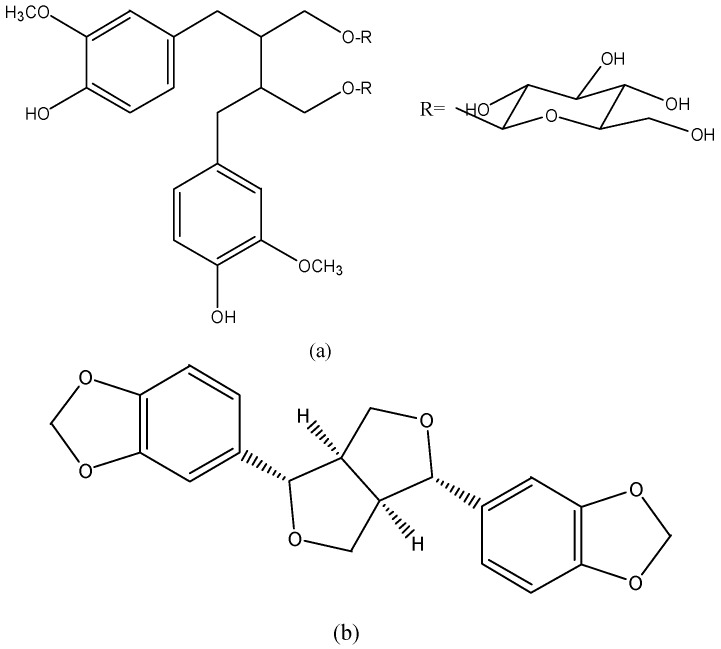
The chemical structures of (**a**) Secoisolariciresinol diglucoside and (**b**) sesamin.

Lignans (Figure 9) are widely present in plants, such as flaxseed, sesame, and seeds of *Arctium lappa*. Secoisolariciresinol diglucoside (SDG) is a natural lignan rich in flaxseed, and can be converted into more biologically active lignans (enterodiol and enterolactone) by human colon bacteria. These lignans are structurally similar to estradiol; thus, they may have anticancer effects for hormone-related cancers, such as breast, prostate and colon cancer. For example, SDG was reported to possess selective estrogen receptor modulating effects and display anti-estrogenic activity in a high estrogen environment. Treatment with SDG (100 ppm in diet) normalized some biomarkers changed by carcinogen in mammary gland tissue of mice [160]. In another study, enterolactone modulated expression of genes involved in cell proliferation and cell cycle of MDA-MB-231 breast cancer cells (IC_50_ 261.9 ± 10.5 μM) [161].

Sesamin is a major lipid soluble lignan from sesame oil. Sesamin treatment (1, 10 and 50 μM) dose-dependently decreased cell viability and increased apoptosis in MCF-7 breast cancer cells. The lignan (10–100 μM) also inhibited the pro-angiogenic activity of macrophages in MCF-7 cells by down-regulating VEGF and MMP-9 [162]. Besides, it was suggested that STAT3 played an important role in sesamin (25–125 μM) induced G_2_/M cell cycle arrest and apoptosis in HepG2 cells [163]. Sesamin (10–100 μg/mL) could suppress lipopolysaccharide-induced proliferation and invasion of PC3 prostate cancer cells by modulating the p38-MAPK and NF-κB signaling pathways. Likewise, sesamin pretreatment (10 mg/kg every three days, injection) suppressed PC3 cells-derived tumor growth triggered by lipopolysaccharide in mice [164]. 

### 3.10. Stilbenes

**Figure 10:**
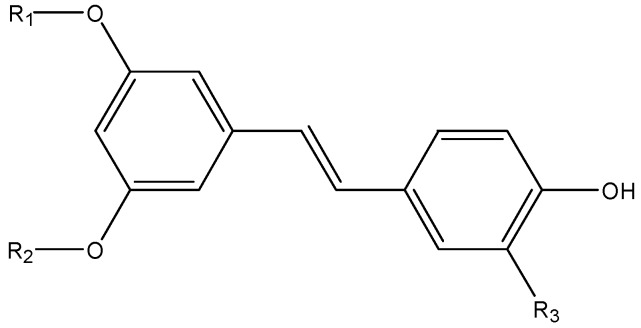
The chemical structures of resveratrol (R_1_ = R_2_ = R_3_ = H), pterostilbene (R_1_ = R_2_ = CH3, R_3_ = OH), piceatannol (R_1_ = R_2_ = H, R_3_ = OH).

Natural stilbenes (Figure 10) are another important group of polyphenols. Though they only exist in a limited group of plant families, the prominent health benefits of resveratrol, an important member of this class, have attracted a lot of studies into natural stilbenes.

#### 3.10.1. Resveratrol

Resveratrol is predominantly found in red wine, grapes and berries. X-ray repair cross complement group 1 (XRCC1) participates in base excision repair. It was reported that resveratrol treatment (5–50 μM) could suppress XRCC1 expression, thus leading to enhanced chemosensitivity to etoposide (a topoisomerase II inhibitor) of human non-small-cell lung cancer cell lines [165]. Besides, it was reported that 20 μM resveratrol treatment significantly suppressed invasion and metastasis of A549 lung cancer cells by inhibiting EMT [11]. 

In gastric cancer cells, resveratrol treatment (25 and 50 μM) arrested cancer cells in the G_1_ phase, resulting in senescence instead of apoptosis. In similar ways, resveratrol (40 mg/kg/day) inhibited gastric cancer development in nude mice [166]. However, at higher concentrations (50–200 μM), resveratrol induced DNA damage and apoptosis in human gastric adenocarcinoma cells via promoting generation of ROS [167]. Resveratrol induced apoptosis in different colon cancer cell lines via modulating diverse targets. For example, resveratrol induced caspase-8 and -3 dependent apoptosis via ROS-triggered autophagy in HT-29 (IC_50_ 150 μM) and COLO 201 (IC_50_ 75 μM) human colon cancer cells [168]. A study reported that the indirect DNA-damaging effects of resveratrol (30 μM) in colon cancer cells were mainly caused by overproduction of ROS [169]. Another study suggested that the DNA damage induced by resveratrol (25 μM) was due to topoisomerase II poisoning rather than promoting ROS production [170]. Besides, resveratrol (50 μM) suppressed expression of multi-drug resistance protein 1 (MDR1) and drug efflux in drug-resistant colorectal cancer cells [171]. Activating mutations in Kras contribute to sporadic colorectal cancer. An in vivo study found that dietary supplements of resveratrol (equivalent to 105 and 210 mg daily for humans) protected against formation and growth of colorectal cancer by suppressing expression of Kras [172]. In addition, in colorectal cancer patients, following oral administration of resveratrol, high concentrations of resveratrol conjugates (mainly RSV-3-*O*-sulfate, RSV-3-*O*-glucuronide and RSV-4′-*O*-glucuronide) were found in the colorectum. Mixture of these conjugates exhibited synergistic anticancer effects by inducing DNA damage and apoptosis in human colorectal cancer cells. Therefore, despite the low bioavailability of resveratrol, the anti-carcinogenic properties could also be achieved by its main metabolites [173]. Cancer stem cells possess the ability to self-renew and are important for tumor generation. Three signaling pathways regulated the self-renewal of breast cancer stem cells are Wnt, Notch and Hedgehog. It was reported that resveratrol could inhibit the Wnt/β-catenin signaling pathway in breast cancer stem cells. Accordingly, resveratrol treatment (i.v. 100 mg/kg/day) to mice significantly suppressed tumor growth as well as the breast cancer stem cells in primary xenografts [174]. Resveratrol is also a powerful chemopreventive agent against liver cancer. At low concentration, resveratrol treatment (25–100 μM) inhibited metastasis of HCC cells and decreased expression of urokinase-type plasminogen activator (u-PA), which involved down-regulation of the SP-1 signaling pathway [175]. Besides, in *N*-nitrosodiethylamine treated rat, the oral administration of resveratrol (20 mg/kg/day) either at early or advanced stages of liver carcinogenesis was equally effective, possibly mediated by apoptosis [176]. In androgen independent prostate cancer cells, resveratrol treatment (25–100 μM) induced autophagy-mediated cell death [177]. In addition, oral administration of resveratrol (30 mg/kg thrice a week) to mice inhibited proliferation, induced apoptosis, and suppressed angiogenesis and metastasis of prostate cancer [178]. In several cervical cancer cells, resveratrol treatment (150–250 μM) caused cell cycle arrest and apoptosis [179].

#### 3.10.2. Pterostilbene

Pterostilbene is a natural dimethoxylated analog of resveratrol mainly found in blueberries. The hydroxyl group substitution with methoxyl groups gives pterostilbene greater lipophilicity, oral bioavailability and biological half-life than resveratrol. 

Pinostilbene is a major metabolite of pterostilbene in the colon of mice. At physiologically relevant concentrations (20 and 40 μM), it significantly inhibited cell growth, and induced apoptosis and S phase arrest of human colon cancer cells. Therefore, pinostilbene might be important for the anticancer effects of orally administered pterostilbene [180]. In addition, pterostilbene treatment (25–75 μM) was able to induce apoptosis in breast cancer cells via Bax activation and over-expression [181]. MicroRNAs (miRNAs) are small non-coding RNAs, which control post-transcriptional expression of genes. It was suggested that miRNAs are highly involved in the development of cancer [48]. A study reported that pterostilbene treatment inhibited EMT and metastasis of breast cancer cells (2.5–10 μM). Mechanistic investigations also showed an up-regulation of miR-205 following pterostilbene treatment, which inhibited the Src/Fak signaling and suppressed tumor growth and metastasis in MDA-MB-231-bearing NOD/SCID mice (i.p. 10 mg/kg thrice a week) [182]. Another study found that pterostilbene treatment selectively killed breast cancer stem cells (IC_50_ 25 μM) and sensitized these cells to chemotherapeutic drug paclitaxel [183]. Besides, pterostilbene treatment (80 μM) activated AMPK in both p53 positive and negative human prostate cancer cells, but the cell fate following AMPK activation was affected by p53 status. In p53 positive LNCaP cells, pterostilbene caused G_1_ cell cycle arrest by increasing p53 expression, while in p53 negative PC3 cells, pterostilbene treatment induced apoptosis [184]. In another study, pterostilbene (i.p. 50 mg/kg/day) inhibited tumor growth in mice models of prostate cancer [185].

#### 3.10.3. Piceatannol

Piceatannol is a hydroxylated analog of resveratrol present in a variety of foods, for example, grapes, berries, passion fruit, and white tea. In colorectal cancer cells, piceatannol treatment (30 μM) induced apoptosis by up-regulating miR-129, and thus down-regulating Bcl-2, which is a known target of miR-129 [186]. Besides, in prostate cancer cells, treatment with piceatannol (25 and 50 μM) inhibited proliferation, and induced cell cycle arrest and apoptosis, which might be associated with down-regulated mTOR [187]. Piceatannol was also a potential anti-invasive and anti-metastasis agent on prostate cancer cells. The oral administration of piceatannol (20 mg/kg/day) significantly suppressed the metastasis of prostate cancer to lung in mice [188].

**Figure 11:**
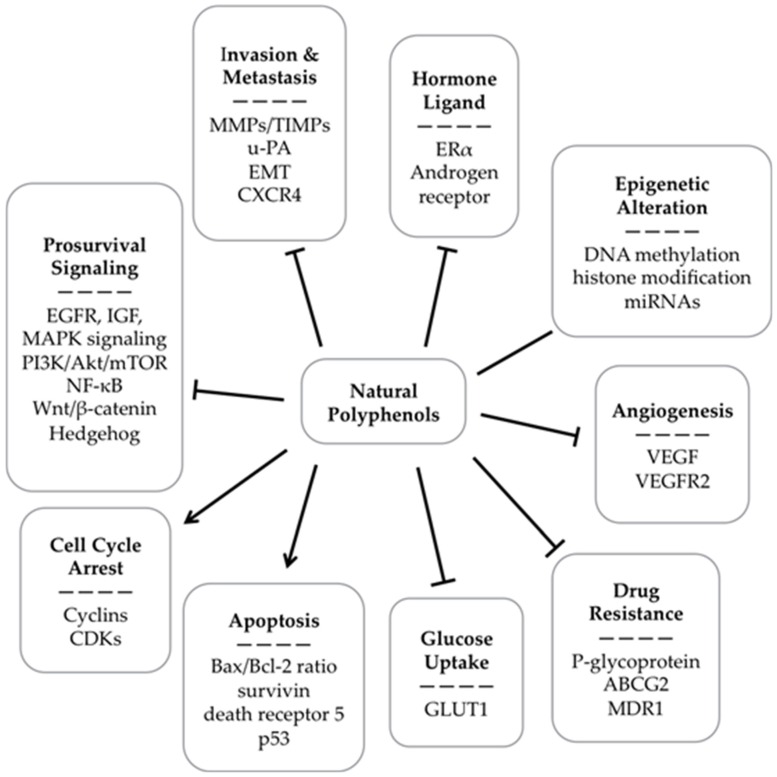
Mechanisms of the anticancer activities of natural polyphenols → stands for activation, – for regulation, ⊥ for inhibition.

The anticancer activities and potential mechanisms of the polyphenols reviewed in this section were summarized in Table 3 and Figure 11. Due to the critical role of cancer stem cells in cancer development and treatment, the anti-cancer stem cell effects of polyphenols were summarized in Table 4. It should be noted that curcumin is not discussed in this section because it has been extensively reviewed [189,190,191]. Besides, the bioavailability of many polyphenols is low, which might hamper their application in cancer treatment (Table 5) [6].

## 4. Clinical Trials

Though numerous studies have demonstrated that natural polyphenol could be potential candidates for anticancer therapy, clinical studies in this area are relatively few and the therapeutic efficacy is sometimes non-significant. A review of early clinical investigations on polyphenolic phytochemicals suggested tea polyphenols could be used for the prevention of premalignancy, but evidence was less convincing for curcumin and soy isoflavones [192]. Table 6 summarized some clinical evidence about the use of natural polyphenol in cancer treatment. The clinical trials in this section were identified from the PubMed database using the MeSH term “neoplasms” combined with “polyphenols”.

## 5. Conclusions

The epidemiological studies about the relationship between dietary polyphenol consumption and cancer risks yielded different results. The difficult in assessing intake of dietary polyphenols and the diversity of polyphenols might contribute to the inconsistent results. On the other hand, the vast majority of laboratory studies supported anticancer activities of natural polyphenols, such as anthocyanins, EGCG, resveratrol and curcumin. The mechanisms of action mainly included modulation of molecular events and signaling pathways associated with cell survival, proliferation, differentiation, migration, angiogenesis, hormone activities, detoxification enzymes, immune responses, etc. Besides, the anticancer effects of polyphenol varied with cancer types, cell lines and doses. It is of note that some polyphenols, such as genistein and daidzein, have been suggested to have adverse effects on hormone-related cancer. Therefore, the use of these polyphenols in cancer treatment should be cautious. In addition, clinical trials about the anticancer actions of polyphenol are limited. In the future, more epidemiological studies employing biomarkers of polyphenols are needed to assess the impact of dietary polyphenols on cancer risks. Besides, the anticancer activities of more polyphenols need to be assessed and compared, and the mechanisms of action require further study. Larger, randomized clinical trials need to be carried out to provide more reliable evidence. Additionally, the bioavailability of polyphenols should be evaluated and improved. Special attention should be paid to the safety of polyphenols.

## Figures and Tables

**Table 1:** The classification of natural polyphenols.

Classification		Representative Members	Major Dietary Sources
flavonoids	anthocyanins	delphinidin, pelargonidin, cyanidin, malvidin	berries, grapes, cherries, plums, pomegranates
	flavanols	epicatechin, epigallocatechin, EGCG, procyanidins	apples, pears, legumes, tea, cocoa, wine
	flavanones	hesperidin, naringenin	citrus fruits
	flavones	apigenin, chrysin, luteolin,	parsley, celery, orange, onions, tea, honey, spices
	flavonols	quercetin, kaempferol, myricetin, isorhamnetin, galangin	berries, apples, broccoli, beans, tea
	isoflavonoids	genistein, daidzein	soy
phenolic acids	hydroxybenoic acid	ellagic acid, gallic acid	pomegranate, grapes, berries, walnuts, chocolate, wine, green tea
	hydroxycinnamic acid	ferulic acid, chlorogenic acid	coffee, cereal grains
lignans		sesamin, secoisolariciresinol diglucoside	flaxseeds, sesame
stilbenes		resveratrol, pterostilbene, piceatannol	grapes, berries, red wine

**Table 2:** Dietary polyphenol intake and cancer risks.

Cancer	Polyphenols	Study Type	Risk Estimates (95% CI)	References
lung cancer	flavonoids	case-control study	0.63 (0.47–0.85)	[14]
gastric cancer	flavonoids	case-control study	no significant association	[16]
	flavonoids	case-control study	0.33 (0.15–0.73)	[15]
colorectal cancer	flavonoids	cohort study	no significant association	[19]
	flavonoids and lignans	case-control study	total flavonoids 0.59 (0.35–0.99); lignans 0.59 (0.34–0.99)	[18]
	polyphenols	case-control study	no significant association	[20]
	isoflavones	meta-analysis	0.76 (0.59–0.98)	[17]
HCC	flavanols	cohort study	0.62 (0.33–0.99)	[22]
breast cancer	flavonoids	meta-analysis	flavonols 0.88 (0.80–0.98); flavones 0.83 (0.76–0.91); no significant association for total flavonoids or other subclasses	[23]
	isoflavones	meta-analysis	0.68 (0.52–0.89)	[25]
	flavanols	cohort study	0.81 (0.67–0.97)	[26]
prostate cancer	flavonoids	cohort study	1.15 (1.04–1.27)	[28]
	flavonoids	cohort study	total catechin 0.73 (0.57–0.95); epicatechin 0.74 (0.57–0.95); kaempferol 0.78 (0.61–1.00); myricetin 0.71 (0.55–0.91)	[27]

**Table 3:** The in vitro and in vivo anticancer activities of natural polyphenols.

Polyphenol	Study Type	Dose	Main Effects	References
**Lung Cancer**				
peonidin-3-glucoside	in vitro	10–40 μM	inhibiting cancer cell invasion, motility, secretion of MMPs and u-PA	[33]
anthocyanidins	in vivo	0.5 mg/mouse	inhibiting tumor growth	[7]
xanthohumol	in vitro	14–42 μM	inducing apoptosis and cell cycle arrest	[38]
EGCG	in vitro	5–20 μM	suppressing cancer cell invasion, migration, MMP-2	[44]
EGCG	in vivo	NA ^1^	suppressing nicotine-induced angiogenesis	[9]
procyanidin C1	in vitro	1.25–40 μg/mL	inhibiting TGF-β-induced EMT	[56]
naringenin	in vitro	100 μM	enhancing TRAIL-mediated apoptosis	[60]
apigenin	in vitro	40–160 μM	inducing apoptosis and DNA damage	[74]
chrysin	in vitro	10 μM	inducing apoptosis, AMPK activation, ROS	[83]
luteolin	in vitro	5–50 μM	inducing apoptosis, cell cycle arrest, inhibiting monocyte recruitment, migration, EMT	[85,86,87]
luteolin	in vivo	10–30 mg/kg	suppressing tumor growth	[88]
quercetin	in vivo	8.4 mg/kg	suppressing tumor growth	[95]
kaempferol	in vitro	10–50 μM	inhibiting TGF-β1-induced EMT and migration	[107]
isorhamnetin	in vivo	NA	suppressing tumor growth	[123]
genistein	in vitro	25–75 μM	suppressing cancer cell proliferation and migration, accompanied by apoptosis and cell cycle arrest	[130]
resveratrol	in vitro	5–50 μM	decreasing XRCC1 expression, enhancing chemosensitivity, suppressing invasion, metastasis	[13,165]
**Gastric Cancer**				
EGCG	in vitro	20–100 μM	inducing apoptosis, down-regulating survivin, the β-catenin signaling pathway	[45,46]
naringenin	in vitro	20–80 μM	inducing apoptosis, inhibiting cancer cell proliferation, invasion, migration and the AKT pathway	[61]
hesperetin	in vivo	20–40 mg/kg	suppressing tumor growth	[67]
apigenin	in vitro	20 μg/mL	inducing apoptosis	[75]
apigenin	in vivo	30–60 mg/kg	preventing *Helicobacter pylori*-induced atrophic gastritis and carcinogenesis	[76]
luteolin	in vivo	10 mg/kg	suppressing tumor growth	[89]
quercetin	in vitro	40–160 μM	inducing apoptosis and protective autophagy	[96]
kaempferol	in vivo	20 mg/kg	suppressing tumor growth	[108]
myricetin	in vitro	20–40 μM	inducing apoptosis and cell cycle arrest	[115]
galangin	in vitro	50–200 μM	inducing apoptosis	[118]
isorhamnetin	in vivo	1 mg/kg	increasing PPAR-γ, decreasing Bcl-2 and CD31	[124]
gallic acid	in vivo	0.25% and 0.5% in water	decreasing tumor size and weight	[151]
resveratrol	in vitro	50–200 μM	inducing apoptosis, DNA damage, ROS production	[167]
resveratrol	in vivo	40 mg/kg	suppressing tumor growth	[166]
**Colorectal Cancer**				
delphinidin	in vitro	30–240 μM	inducing apoptosis, cell cycle arrest, oxidative stress	[30]
cyanidin	in vitro	100 μM	inducing oxidative stress	[35]
EGCG	in vitro	1–50 μM	inducing epigenetic alteration, apoptosis, MAPK and Akt pathways activation	[47,49]
procyanidins	in vitro	50 and 100 μM	inducing apoptosis and cell cycle arrest	[57]
naringenin	in vitro	50–200 μM	inducing apoptosis	[62]
hesperetin	in vivo	20 mg/kg	suppressing chemical-induced carcinogenesis	[68]
apigenin	in vivo	50 mg/kg	inhibiting tumor growth and metastasis	[77]
chrysin	in vitro	50–100 μM	inducing TNF-mediated apoptotic cell death	[84]
luteolin	in vitro	20-100 μM	inducing apoptosis and cell cycle arrest	[90]
quercetin	in vivo	25–50 mg/kg	suppressing tumor growth by reducing AMPK activity and alleviating cachexia symptoms	[97,98]
kaempferol	in vitro	0–60 μM	inducing apoptosis	[109]
myricetin	in vitro	NA	inducing apoptosis	[116]
isorhamnetin	in vivo	200 g/kg in diet	suppressing mortality, tumor number, tumor burden and chemical-induced inflammatory responses	[126]
genistein	in vivo	20–80 mg/kg	decreasing the weight and size of transplanted tumor, inhibiting angiogenesis and metastasis	[132,133]
ellagic acid	in vitro	50–200 μM	inducing apoptosis	[144]
gallic acid	in vitro	200 μM	inducing apoptosis	[149]
resveratrol	in vitro	25–150 μM	inducing apoptosis, DNA damage and suppressing drug resistance	[168,169,170,171]
resveratrol	in vivo	equal to 105 and 210 mg for human	suppressing tumor development by modulation of Kras	[172]
piceatannol	in vitro	30 μM	inducing apoptosis mediated by miR-129	[186]
**Liver Cancer**				
xanthohumol	in vitro	5–40 μM	inducing apoptosis, modulating the NF-κB/p53 and the Notch1 signaling pathways	[39,40]
naringenin	in vitro	25–200 μM	suppressing TPA-induced cancer cell invasion, inducing apoptosis and cell cycle arrest	[63,64]
quercetin	in vivo	40 mg/kg	suppressing tumor growth	[99]
kaempferol	in vitro	25–100 μM	inducing cell cycle arrest and autophagy	[110]
myricetin	in vivo	100 mg/kg	suppressing chemical-induced carcinogenesis	[117]
galangin	in vitro	10–134 μM	inhibiting chemical-induced cell invasion, metastasis, promoting ER stress	[119,120]
daidzein	in vitro	200–600 μM	inducing apoptosis	[127]
genistein	in vivo	50 mg/kg	suppressing the intrahepatic metastasis	[136]
ellagic acid	in vivo	30 mg/kg	suppressing chemical-induced carcinogenesis	[145]
gallic acid	in vitro	22.1–28.5 μg/mL	inducing apoptosis	[153]
sesamin	in vitro	25–125 μM	inducing apoptosis and cell cycle arrest mediated by STAT3	[163]
resveratrol	in vitro	25–100 μM	inhibiting metastasis, decreasing expression of u-PA, down-regulating the SP-1 signaling pathway	[175]
resveratrol	in vivo	20 mg/kg	suppressing chemical-induced carcinogenesis	[176]
**Breast Cancer**				
anthocyanins	in vivo	6 mg/kg	suppressing the growth of HER2-positive tumor	[32]
cyanidin-3-*O*-sambubioside	in vitro	1–30 μM	inhibiting angiogenesis and invasion	[34]
xanthohumol	in vitro	NA	decreasing expression of CXCR4, inhibiting cell invasion induced by CXCL12	[42]
xanthohumol	in vivo	0.3 and 1.0 mg/kg	blocking the estrogen singling pathway, selectively suppressing the growth of ERα-positive breast cancer	[41]
EGCG	in vitro	1–40 μM	suppressing estrogen-induced cancer cell proliferation, down-regulating ERα , inhibiting metastasis by restoring the balance between MMP and TIMP	[52,53,54]
procyanidins	in vitro	25–100 μM	inducing apoptosis, reducing invasion, angiogenesis	[59]
naringenin	in vivo	100 mg/kg	suppressing lung metastases by the host immunity	[66]
hesperetin	in vitro	40–200 μM	inducing apoptosis, ROS production and activation of ASK1/JNK pathway, suppressing glucose uptake	[69,70]
apigenin	in vitro	20–100 μM	suppressing growth and causing apoptosis possibly mediated by the STAT3 signaling pathway	[78]
apigenin	in vivo	5–25 mg/kg	inducing cell cycle arrest through epigenetic change	[79]
chrysin	in vitro	5–20 μM	inhibiting cancer cell invasion and migration	[84]
luteolin	in vitro	10–40 μM	down-regulating ERα expression, inducing apoptosis and cell cycle arrest	[92]
luteolin	in vivo	0.01%–0.05% in diet	reducing tumor burden	[93]
quercetin	in vitro	1–200 μM	inducing apoptosis, suppressing the insulin receptor signaling and EMT	[100,101,102]
quercetin	in vivo	34 mg/kg	inhibiting angiogenesis	[103]
kaempferol	in vitro	100 μM	inducing apoptosis and suppressing glucose uptake	[111,112]
kaempferol	in vivo	100 mg/kg	preventing cancer development induced by estrogen	[113]
isorhamnetin	in vitro	10–40 μM	inhibiting cancer cell adhesion, migration, invasion	[122]
daidzein	in vitro	3–50 μM	decreasing invasion, MMP-2 expression, up-regulating proto-oncogene BRF2 in ER-positive cancer cells	[128,129]
genistein	in vitro	5–20 μM	inducing apoptosis, cell cycle arrest, increasing drug resistance	[10,137]
genistein	in vivo	≤500 ppm	enhancing tumor growth	[140]
ellagic acid	in vitro	10–40 μg/mL	inducing cell cycle arrest	[143]
ellagic acid	in vivo	50–100 mg/kg	suppressing tumor growth and angiogenesis	[146]
gallic acid	in vitro	80.5 µM	inducing apoptosis	[150]
SDG	in vivo	100 ppm in diet	normalizing some biomarkers changed by carcinogen	[160]
enterolactone	in vitro	261.9 ± 10.5 μM	modulating expression of genes involved in cell proliferation and cell cycle	[161]
sesamin	in vitro	1–100 μM	inducing apoptosis and inhibiting the pro-angiogenic activity of macrophages	[162]
pterostilbene	in vitro	25–75 μM	inducing apoptosis	[181]
pterostilbene	in vivo	10 mg/kg	suppressing tumor growth and metastasis	[182]
**Prostate Cancer**				
delphinidin	in vitro	3–90 μM	inducing apoptosis and cell cycle arrest	[31]
xanthohumol	in vivo	50 μg/mouse	suppressing tumor growth and progression	[43]
EGCG	in vivo	1 mg 3×/week	antagonizing androgen, suppressing tumor growth	[55]
hesperetin	in vitro	40–90 μM	inducing apoptosis, inhibiting the NF-κB pathway	[71]
apigenin	in vivo	20 and 50 μg/mouse	suppressing tumor growth, angiogenesis, metastasis	[81]
luteolin	in vitro	30 μM	inducing apoptosis, cell cycle arrest, inhibiting invasion	[94]
quercetin	in vivo	200 mg/kg	inhibiting carcinogenesis induced by hormone and carcinogen	[104]
genistein	in vitro	0.5–50 μM	different effects dependent on androgen receptor	[141]
ellagic acid	in vitro	10–100 μM	inducing apoptosis, inhibiting cell invasion, motility	[147,148]
gallic acid	in vitro	25–200 μM	provoking DNA damage, down-regulating DNA repair genes, invasion and migration	[154,155]
ferulic acid	in vitro	300–500 μM	inducing apoptosis and cell cycle arrest	[158]
sesamin	in vivo	10 mg/kg	suppressed tumor growth induced by LPS	[164]
resveratrol	in vitro	25–100 μM	inducing autophagy-mediated cell death	[177]
resveratrol	in vivo	30 mg/kg	inducing apoptosis, suppressing angiogenesis and metastasis	[178]
pterostilbene	in vitro	80 μM	inducing apoptosis and cell cycle arrest	[184]
pterostilbene	in vivo	50 mg/kg	suppressing tumor growth	[185]
piceatannol	in vitro	25 and 50 μM	inducing apoptosis and cell cycle arrest	[187]
piceatannol	in vivo	20 mg/kg	suppressing lung metastasis	[188]
**Cervical Cancer**				
hesperetin	in vitro	650 μM	inducing apoptosis	[72]
quercetin	in vitro	110.38 μM	inducing apoptosis and cell cycle arrest	[106]
genistein	in vitro	100 μM	inducing apoptosis, cell cycle arrest, suppressing cell migration	[142]
gallic acid	in vitro	10–40 μg/mL	decreasing cell proliferation, invasion, angiogenesis	[156]
ferulic acid	in vitro	10 μg/mL	enhancing efficacy of radiotherapy	[159]
resveratrol	in vitro	150–250 μM	inducing apoptosis and cell cycle arrest	[179]

^1^ NA, stands for not available.

**Table 4:** The anti-cancer stem cell effects of polyphenols.

Compound	Cancer	Study Type	Dose	Effect	References
EGCG	colorectal cancer	in vivo	100 μM	Inhibiting tumor growth of spheroid-derived cancer stem cell xenografts	[50]
	breast cancer	in vivo	16.5 mg/kg	decreasing tumor growth, the expression of VEGF-D and peritumoral lymphatic vessel density	[51]
genistein	gastric cancer	in vivo	1.5 mg/kg	decreasing tumor weight and size	[131]
	breast cancer	in vivo	20–50 mg/kg	targeting breast cancer stem cells to reduce the growth of xenograft tumors and inhibiting the Hedgehog-Gli1 signaling pathway	[138]
resveratrol	breast cancer	in vivo	100 mg/kg	inhibited the Wnt/β-catenin signaling pathway, tumor growth and cancer stem cells	[174]
pterostilbene	breast cancer	in vitro	25 μM	decreasing cancer stem cells and drug resistance	[183]

**Table 5:** The bioavailability of some natural polyphenols.

Compound	Subject	Treatment	Urine Concentration	Plasm Concentration
anthocyanins	human	black berries 200 g (960 µmol) *	total urinary excretion of anthocyanin metabolites 0.160%	NA
EGCG	human	2 mg/kg	NA	mean Cmax 0.09 µmol/L, Tmax 2 h
naringenin	human	fresh orange segments 150 g (11.8 mg/150 g fresh weight) *	mean urinary excretion 12.5%	mean Cmax 0.08 µmol/L, Tmax 5.88 h
hesperetin	human	fresh orange segments 150 g (79.7 mg/150 g fresh weight) *	mean urinary excretion 4.53%	mean Cmax 0.09 µmol/L, Tmax 7 h
quercetin	human	dry shallot skin 1.4 mg/kg (4.93 µmol/g fresh weight) *	NA	mean Cmax 3.95 µmol/L, Tmax 2.78 h
isorhamnetin	rat	0.25 mg/kg	NA	mean Cmax 0.18 µmol/L, Tmax 8 h
daidzein	human	soy milk 750 mL/day (5.4 mg/250 mL) *	148.35 µmol/24 h after 5 days	196.1 nmol/L after 5 days
genistein	human	soy milk 750 mL/day (16.98 mg/250 mL) *	2077.7 µmol/24 h after 5 days	797.04 nmol/L after 5 days
ellagic acid	human	freeze-dried black raspberry 45 g/day (0.3 mg/g dry weight) *	NA	mean Cmax 0.01 µmol/L, Tmax 1.98 h
gallic acid	human	grape skin extract 18 g (0.7 mg/g dry weight) *	5.9 µmol after 24 h	NA
ferulic acid	rat	5.15 mg/kg	mean urinary excretion 43.4%	mean Cmax 1.68 µmol/L, Tmax 1 h
resveratrol	human	1 mg/kg trans-resveratrol	mean urinary excretion 26%	0.75 µg/mL after 1.5 h

* Indicates content of the compound in food; NA, stands for not available.

**Table 6:** Summary of clinical trials with polyphenols in various cancers.

Subject	Treatment	Outcome	References
54 patients with localized prostate cancer	synthetic genistein (30 mg) daily for 3–6 weeks	decreasing level of serum prostate specific antigen (PSA)	[193]
158 men aged 50–75 with rising prostate specific antigen	isoflavone (60 mg) daily for 12 months	reducing prostate cancer incidence for patients aged 65 or more	[194]
86 patients with localized prostate cancer	soy isoflavone (80 mg total isoflavones, 51 mg aglucon units) daily for 6 weeks	no significant change in serum hormone levels, total cholesterol, or PSA	[195]
10 breast cancer patients undergoing radiotherapy	EGCG (400 mg) thrice daily for 2–8 weeks	enhancing efficacy of radiotherapy	[196]
147 patients with prostate cancer	flaxseed (30 mg) daily for 30 days	significant inverse association between total urinary enterolignans and enterolactone and Ki67 in the tumor tissue	[197]
87 patients with resected colorectal cancer or polypectomy	flavonoid mixture (20 mg apigenin and 20 mg EGCG) for 3–4 years	reducing recurrence rate of colon neoplasia in patients with resected colon cancer	[198]
5 familial adenomatous polyposis patients with colectomy	curcumin (480 mg) and quercetin (20 mg) thrice daily for 6 months	reducing polyp number and size from baseline without appreciable toxicity	[199]
85 patients with prostate cancer	isoflavones (40 mg) and curcumin (100 mg) daily for 6 months	decreasing level of serum PSA	[200]
44 smokers with 8 or more aberrant crypt foci	curcumin (2 or 4 g) daily for 30 days	decreasing number of aberrant crypt foci	[201]
126 patients with colorectal cancer	curcumin (360 mg) thrice daily for 10–30 days	increasing body weight and expression of p53, suppressing serum level of TNF-α	[202]
